# [*N*′-(3,5-Diiodo-2-oxidobenzyl­idene-κ*O*)-4-methyl­benzohydrazidato-κ^2^ *N*′,*O*](methanol-κ*O*)(methano­lato-κ*O*)oxidovanadium(V)

**DOI:** 10.1107/S1600536811010385

**Published:** 2011-03-26

**Authors:** Lin Liu

**Affiliations:** a College of Chemistry and Biology Engineering, Yichun University, Yichun 336000, People’s Republic of China

## Abstract

In the title mol­ecule, [V(C_15_H_10_I_2_N_2_O_2_)(CH_3_O)O(CH_3_OH)], the V^V^ atom is coordinated by one N and two O atoms from an *N*′-(3,5-diiodo-2-oxidobenzyl­idene-κ*O*)-4-methyl­benzo­hydra­zidate (*L*) ligand, one oxide O atom, one methano­late [V—O = 1.761 (3) Å] and one methanol [V—O = 2.383 (4) Å] O atom in a distorted octa­hedral geometry. In the *L* ligand, the two benzene rings are nearly parallel, forming a dihedral angle of 2.0 (1)°. In the crystal, inter­molecular O—H⋯N hydrogen bonds link pairs of mol­ecules into centrosymmetric dimers which exhibit π–π inter­actions between the aromatic rings [centroid–centroid distance = 3.677 (5) Å].

## Related literature

For background to oxidovanadium complexes, see: Chohan *et al.* (2010 ▶); Chohan & Sumrra (2010 ▶); Sharma *et al.* (2010 ▶); Tian *et al.* (2010 ▶). For similar oxidovanadium(V) complexes, see: Wang (2011 ▶); Rajak *et al.* (2000 ▶); Mondal *et al.* (2009 ▶).
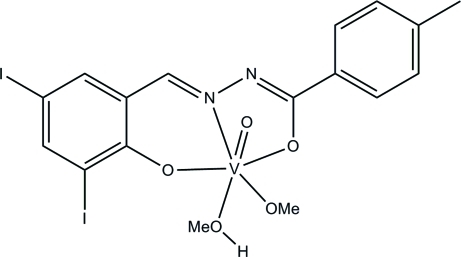


## Experimental

### 

#### Crystal data

[V(C_15_H_10_I_2_N_2_O_2_)(CH_3_O)O(CH_4_O)]
*M* *_r_* = 634.07
Triclinic, 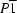

*a* = 7.890 (5) Å
*b* = 10.030 (6) Å
*c* = 13.628 (8) Å
α = 81.857 (5)°
β = 84.777 (6)°
γ = 85.286 (5)°
*V* = 1060.5 (11) Å^3^
*Z* = 2
Mo *K*α radiation
μ = 3.41 mm^−1^
*T* = 298 K
0.17 × 0.13 × 0.12 mm

#### Data collection

Bruker SMART CCD area-detector diffractometer
Absorption correction: multi-scan (*SADABS*; Sheldrick, 1996 ▶) *T*_min_ = 0.595, *T*_max_ = 0.685
7706 measured reflections
4283 independent reflections
3146 reflections with *I* > 2σ(*I*)
*R*_int_ = 0.026

#### Refinement

*R*[*F* ^2^ > 2σ(*F* ^2^)] = 0.041
*wR*(*F* ^2^) = 0.090
*S* = 1.02
4283 reflections
250 parameters
1 restraint
H atoms treated by a mixture of independent and constrained refinement
Δρ_max_ = 1.00 e Å^−3^
Δρ_min_ = −1.01 e Å^−3^

### 

Data collection: *SMART* (Bruker, 1998 ▶); cell refinement: *SAINT* (Bruker, 1998 ▶); data reduction: *SAINT*; program(s) used to solve structure: *SHELXS97* (Sheldrick, 2008 ▶); program(s) used to refine structure: *SHELXL97* (Sheldrick, 2008 ▶); molecular graphics: *SHELXTL* (Sheldrick, 2008 ▶); software used to prepare material for publication: *SHELXTL*.

## Supplementary Material

Crystal structure: contains datablocks global, I. DOI: 10.1107/S1600536811010385/cv5063sup1.cif

Structure factors: contains datablocks I. DOI: 10.1107/S1600536811010385/cv5063Isup2.hkl

Additional supplementary materials:  crystallographic information; 3D view; checkCIF report

## Figures and Tables

**Table 1:** Hydrogen-bond geometry (Å, °)

*D*—H⋯*A*	*D*—H	H⋯*A*	*D*⋯*A*	*D*—H⋯*A*
O4—H4⋯N2^i^	0.85 (4)	2.03 (5)	2.858 (5)	168 (8)

Symmetry code: (i) 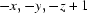.

**Fig. 1.**
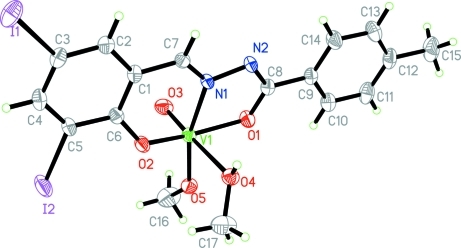
Molecular structure of the title complex showing the atomic numbering and 30% probability ellipsoids.

**Fig. 2.**
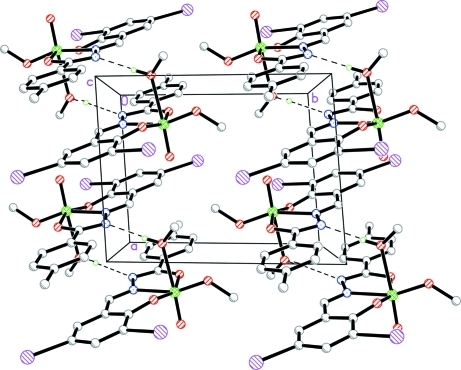
A portion of the crystal packing of (I) viewed approximately down the c axis and showing hydrogen-bonded (dashed lines) dimers.

## supplementary crystallographic information

### Comment

Considerable attention has been focused on the synthesis, structures, and biological properties of oxovanadium complexes (Chohan *et al.*, 2010; Chohan & Sumrra, 2010; Sharma *et al.*, 2010; Tian *et al.*, 2010). The present paper reports the crystal structure of the title new oxovanadium complex (I).

In (I) (Fig. 1), [OV*L*(OCH_3_)(CH_3_OH)] (H_2_*L* = 3,5-diiodosalicylaldehide (4-methylbenzoyl)hydrazonic acid), the V center is coordinated by one N and two O atoms from *L*, one oxo O atom, and two O atoms from the methoxy [V—O 1.761 (3) Å] and methanol [V—O 2.383 (4) Å] ligands, respectively, in a distorted octahedral geometry. In the ligand *L*, two benzene rings are nearly parallel forming a dihedral angle of 2.0 (1)°. The deviation of the V atom from the least-squares plane defined by the three donor atoms of the hydrazone ligand and the methoxy O atom towards the oxo O atom is 0.311 (2) Å. The bond lengths and bond angles related to the V atom are normal and correspond to those observed in the related compounds (Wang, 2011; Rajak *et al.*, 2000; Mondal *et al.*, 2009).

In the crystal structure (Fig. 2), intermolecular O—H···N hydrogen bonds (Table 1) link two molecules into centrosymmetric dimer which exhibits π-π interaction between the aromatic rings [centroid-to-centroid distance of 3.677 (5) Å].

### Experimental

Equimolar quantities (0.1 mmol each) of 3,5-diiodosalicylaldehyde, 4-methylbenzohydrazide, and VOSO_4_ were mixed and stirred in methanol for 30 min at reflux. After keeping the filtrate in air for a few days, red block crystals were formed.

### Refinement

Atom H4 was located in a difference Fourier map and refined with O—H distance restrained to 0.85 (4) Å, and *U*_iso_(H) = 2*U*_eq_(O). Other H atoms were placed in calculated positions and constrained to ride on their parent atoms, with C—H distances in the range 0.93–0.96 Å, and with *U*_iso_(H) = 1.2*U*_eq_(C) and 1.5*U*_eq_(methyl C).

### Crystal data

**Table:**

[V(C_15_H_10_I_2_N_2_O_2_)(CH_3_O)O(CH_4_O)]	*Z* = 2
*M**_r_* = 634.07	*F*(000) = 604
Triclinic, *P*1	*D*_x_ = 1.986 Mg m^−^^3^
*a* = 7.890 (5) Å	Mo *K*α radiation, λ = 0.71073 Å
*b* = 10.030 (6) Å	Cell parameters from 2583 reflections
*c* = 13.628 (8) Å	θ = 2.7–25.0°
α = 81.857 (5)°	µ = 3.41 mm^−^^1^
β = 84.777 (6)°	*T* = 298 K
γ = 85.286 (5)°	Block, red
*V* = 1060.5 (11) Å^3^	0.17 × 0.13 × 0.12 mm

### Data collection

**Table:**

Bruker SMART CCD area-detector diffractometer	4283 independent reflections
Radiation source: fine-focus sealed tube	3146 reflections with *I* > 2σ(*I*)
graphite	*R*_int_ = 0.026
ω scans	θ_max_ = 26.5°, θ_min_ = 2.1°
Absorption correction: multi-scan (*SADABS*; Sheldrick, 1996)	*h* = −9→9
*T*_min_ = 0.595, *T*_max_ = 0.685	*k* = −12→12
7706 measured reflections	*l* = −17→15

### Refinement

**Table:**

Refinement on *F*^2^	Primary atom site location: structure-invariant direct methods
Least-squares matrix: full	Secondary atom site location: difference Fourier map
*R*[*F*^2^ > 2σ(*F*^2^)] = 0.041	Hydrogen site location: inferred from neighbouring sites
*wR*(*F*^2^) = 0.090	H atoms treated by a mixture of independent and constrained refinement
*S* = 1.02	*w* = 1/[σ^2^(*F*_o_^2^) + (0.03*P*)^2^ + 1.6979*P*] where *P* = (*F*_o_^2^ + 2*F*_c_^2^)/3
4283 reflections	(Δ/σ)_max_ < 0.001
250 parameters	Δρ_max_ = 1.00 e Å^−^^3^
1 restraint	Δρ_min_ = −1.01 e Å^−^^3^

### Special details

**Table:**

Geometry. All e.s.d.'s (except the e.s.d. in the dihedral angle between two l.s. planes) are estimated using the full covariance matrix. The cell e.s.d.'s are taken into account individually in the estimation of e.s.d.'s in distances, angles and torsion angles; correlations between e.s.d.'s in cell parameters are only used when they are defined by crystal symmetry. An approximate (isotropic) treatment of cell e.s.d.'s is used for estimating e.s.d.'s involving l.s. planes.
Refinement. Refinement of *F*^2^ against ALL reflections. The weighted *R*-factor *wR* and goodness of fit *S* are based on *F*^2^, conventional *R*-factors *R* are based on *F*, with *F* set to zero for negative *F*^2^. The threshold expression of *F*^2^ > σ(*F*^2^) is used only for calculating *R*-factors(gt) *etc*. and is not relevant to the choice of reflections for refinement. *R*-factors based on *F*^2^ are statistically about twice as large as those based on *F*, and *R*- factors based on ALL data will be even larger.

### Fractional atomic coordinates and isotropic or equivalent isotropic displacement parameters (Å^2^)

**Table:**

	*x*	*y*	*z*	*U*_iso_*/*U*_eq_	
V1	0.23465 (11)	0.25458 (7)	0.54517 (6)	0.0344 (2)	
I1	0.54984 (5)	−0.39607 (4)	0.86399 (3)	0.06551 (15)	
I2	0.39530 (6)	0.20017 (4)	0.87434 (3)	0.06716 (16)	
N1	0.2307 (5)	0.0559 (4)	0.5077 (3)	0.0311 (9)	
N2	0.1714 (5)	0.0473 (4)	0.4152 (3)	0.0346 (9)	
O1	0.1488 (5)	0.2762 (3)	0.4147 (2)	0.0423 (8)	
O2	0.2580 (5)	0.1637 (3)	0.6730 (2)	0.0428 (8)	
O3	0.4296 (4)	0.2723 (3)	0.5122 (3)	0.0497 (9)	
O4	−0.0568 (5)	0.2139 (3)	0.5921 (3)	0.0439 (8)	
O5	0.1603 (4)	0.4154 (3)	0.5753 (3)	0.0417 (8)	
C1	0.3464 (6)	−0.0661 (4)	0.6552 (3)	0.0348 (11)	
C2	0.4118 (6)	−0.1917 (5)	0.7000 (4)	0.0379 (11)	
H2	0.4156	−0.2667	0.6666	0.046*	
C3	0.4699 (6)	−0.2047 (5)	0.7927 (4)	0.0410 (12)	
C4	0.4695 (7)	−0.0928 (5)	0.8431 (4)	0.0459 (13)	
H4A	0.5129	−0.1016	0.9051	0.055*	
C5	0.4040 (6)	0.0303 (5)	0.7998 (4)	0.0394 (12)	
C6	0.3362 (6)	0.0472 (5)	0.7069 (3)	0.0344 (11)	
C7	0.2817 (6)	−0.0561 (4)	0.5582 (4)	0.0337 (11)	
H7	0.2768	−0.1353	0.5307	0.040*	
C8	0.1344 (6)	0.1705 (5)	0.3713 (4)	0.0353 (11)	
C9	0.0718 (6)	0.1932 (5)	0.2712 (4)	0.0378 (11)	
C10	0.0142 (7)	0.3222 (5)	0.2310 (4)	0.0489 (14)	
H10	0.0172	0.3939	0.2671	0.059*	
C11	−0.0475 (7)	0.3452 (6)	0.1377 (4)	0.0585 (16)	
H11	−0.0874	0.4321	0.1128	0.070*	
C12	−0.0512 (7)	0.2434 (7)	0.0813 (4)	0.0559 (15)	
C13	0.0081 (8)	0.1157 (6)	0.1213 (4)	0.0622 (17)	
H13	0.0079	0.0447	0.0842	0.075*	
C14	0.0677 (8)	0.0905 (6)	0.2149 (4)	0.0521 (14)	
H14	0.1054	0.0031	0.2400	0.062*	
C15	−0.1196 (9)	0.2677 (8)	−0.0205 (5)	0.083 (2)	
H15A	−0.0267	0.2622	−0.0707	0.125*	
H15B	−0.1781	0.3558	−0.0301	0.125*	
H15C	−0.1975	0.2006	−0.0252	0.125*	
C16	0.2549 (9)	0.5282 (6)	0.5792 (6)	0.076 (2)	
H16A	0.3014	0.5199	0.6427	0.114*	
H16B	0.1815	0.6094	0.5702	0.114*	
H16C	0.3462	0.5318	0.5275	0.114*	
C17	−0.1589 (9)	0.2807 (7)	0.6648 (5)	0.075 (2)	
H17A	−0.1085	0.2616	0.7273	0.112*	
H17B	−0.2716	0.2491	0.6724	0.112*	
H17C	−0.1654	0.3763	0.6437	0.112*	
H4	−0.087 (10)	0.134 (3)	0.599 (6)	0.088*	

### Atomic displacement parameters (Å^2^)

**Table:**

	*U*^11^	*U*^22^	*U*^33^	*U*^12^	*U*^13^	*U*^23^
V1	0.0406 (5)	0.0279 (4)	0.0370 (5)	−0.0040 (3)	−0.0089 (4)	−0.0077 (3)
I1	0.0621 (3)	0.0531 (2)	0.0724 (3)	0.00130 (19)	−0.0126 (2)	0.0238 (2)
I2	0.0971 (4)	0.0637 (3)	0.0486 (3)	−0.0046 (2)	−0.0270 (2)	−0.02184 (19)
N1	0.035 (2)	0.032 (2)	0.028 (2)	−0.0051 (16)	−0.0065 (17)	−0.0065 (16)
N2	0.040 (2)	0.036 (2)	0.030 (2)	−0.0071 (17)	−0.0084 (18)	−0.0070 (17)
O1	0.058 (2)	0.0309 (17)	0.040 (2)	−0.0039 (15)	−0.0132 (17)	−0.0030 (14)
O2	0.061 (2)	0.0335 (18)	0.036 (2)	0.0048 (16)	−0.0160 (17)	−0.0096 (14)
O3	0.044 (2)	0.049 (2)	0.060 (2)	−0.0112 (17)	0.0022 (18)	−0.0205 (18)
O4	0.042 (2)	0.044 (2)	0.049 (2)	−0.0068 (17)	−0.0020 (17)	−0.0133 (17)
O5	0.046 (2)	0.0276 (16)	0.054 (2)	−0.0019 (14)	−0.0085 (17)	−0.0089 (15)
C1	0.036 (3)	0.034 (2)	0.034 (3)	−0.008 (2)	−0.007 (2)	0.002 (2)
C2	0.038 (3)	0.036 (3)	0.040 (3)	−0.008 (2)	−0.004 (2)	−0.003 (2)
C3	0.036 (3)	0.041 (3)	0.043 (3)	−0.007 (2)	−0.005 (2)	0.009 (2)
C4	0.046 (3)	0.058 (3)	0.034 (3)	−0.010 (3)	−0.012 (3)	0.003 (2)
C5	0.045 (3)	0.042 (3)	0.034 (3)	−0.008 (2)	−0.010 (2)	−0.006 (2)
C6	0.033 (3)	0.039 (3)	0.032 (3)	−0.009 (2)	−0.002 (2)	−0.006 (2)
C7	0.036 (3)	0.029 (2)	0.038 (3)	−0.005 (2)	−0.005 (2)	−0.006 (2)
C8	0.033 (3)	0.038 (3)	0.035 (3)	−0.008 (2)	−0.003 (2)	0.000 (2)
C9	0.033 (3)	0.048 (3)	0.032 (3)	−0.010 (2)	−0.003 (2)	0.000 (2)
C10	0.050 (3)	0.052 (3)	0.045 (3)	−0.010 (3)	−0.010 (3)	0.000 (3)
C11	0.054 (4)	0.069 (4)	0.050 (4)	−0.011 (3)	−0.016 (3)	0.016 (3)
C12	0.041 (3)	0.089 (5)	0.036 (3)	−0.009 (3)	−0.009 (3)	0.003 (3)
C13	0.076 (5)	0.077 (4)	0.037 (3)	−0.009 (4)	−0.016 (3)	−0.010 (3)
C14	0.065 (4)	0.056 (3)	0.036 (3)	−0.003 (3)	−0.013 (3)	−0.002 (2)
C15	0.075 (5)	0.127 (6)	0.046 (4)	−0.008 (4)	−0.023 (4)	0.008 (4)
C16	0.073 (5)	0.037 (3)	0.122 (6)	−0.015 (3)	−0.007 (4)	−0.021 (3)
C17	0.069 (5)	0.069 (4)	0.087 (5)	−0.008 (3)	0.013 (4)	−0.026 (4)

### Geometric parameters (Å, °)

**Table:**

V1—O3	1.580 (4)	C5—C6	1.402 (6)
V1—O5	1.761 (3)	C7—H7	0.9300
V1—O2	1.865 (3)	C8—C9	1.475 (6)
V1—O1	1.938 (3)	C9—C14	1.372 (7)
V1—N1	2.130 (4)	C9—C10	1.389 (7)
V1—O4	2.383 (4)	C10—C11	1.385 (7)
I1—C3	2.100 (5)	C10—H10	0.9300
I2—C5	2.097 (5)	C11—C12	1.366 (8)
N1—C7	1.286 (6)	C11—H11	0.9300
N1—N2	1.400 (5)	C12—C13	1.381 (8)
N2—C8	1.317 (6)	C12—C15	1.514 (8)
O1—C8	1.302 (5)	C13—C14	1.383 (7)
O2—C6	1.319 (5)	C13—H13	0.9300
O4—C17	1.426 (7)	C14—H14	0.9300
O4—H4	0.85 (4)	C15—H15A	0.9600
O5—C16	1.416 (6)	C15—H15B	0.9600
C1—C2	1.400 (6)	C15—H15C	0.9600
C1—C6	1.413 (6)	C16—H16A	0.9600
C1—C7	1.447 (6)	C16—H16B	0.9600
C2—C3	1.368 (7)	C16—H16C	0.9600
C2—H2	0.9300	C17—H17A	0.9600
C3—C4	1.396 (7)	C17—H17B	0.9600
C4—C5	1.373 (7)	C17—H17C	0.9600
C4—H4A	0.9300		
			
O3—V1—O5	102.77 (17)	N1—C7—C1	123.9 (4)
O3—V1—O2	98.49 (18)	N1—C7—H7	118.1
O5—V1—O2	99.38 (15)	C1—C7—H7	118.1
O3—V1—O1	98.80 (18)	O1—C8—N2	121.8 (4)
O5—V1—O1	96.85 (15)	O1—C8—C9	117.6 (4)
O2—V1—O1	152.99 (14)	N2—C8—C9	120.7 (4)
O3—V1—N1	96.54 (16)	C14—C9—C10	117.8 (5)
O5—V1—N1	159.79 (16)	C14—C9—C8	122.4 (5)
O2—V1—N1	83.42 (14)	C10—C9—C8	119.8 (5)
O1—V1—N1	74.12 (14)	C11—C10—C9	120.7 (5)
O3—V1—O4	176.43 (15)	C11—C10—H10	119.7
O5—V1—O4	80.79 (14)	C9—C10—H10	119.7
O2—V1—O4	81.04 (15)	C12—C11—C10	121.7 (6)
O1—V1—O4	80.42 (14)	C12—C11—H11	119.2
N1—V1—O4	79.89 (13)	C10—C11—H11	119.2
C7—N1—N2	116.4 (4)	C11—C12—C13	117.3 (5)
C7—N1—V1	127.8 (3)	C11—C12—C15	121.9 (6)
N2—N1—V1	115.7 (3)	C13—C12—C15	120.8 (6)
C8—N2—N1	108.5 (4)	C12—C13—C14	121.8 (6)
C8—O1—V1	119.9 (3)	C12—C13—H13	119.1
C6—O2—V1	133.0 (3)	C14—C13—H13	119.1
C17—O4—V1	123.2 (3)	C9—C14—C13	120.7 (5)
C17—O4—H4	107 (5)	C9—C14—H14	119.7
V1—O4—H4	119 (6)	C13—C14—H14	119.7
C16—O5—V1	128.6 (4)	C12—C15—H15A	109.5
C2—C1—C6	119.8 (4)	C12—C15—H15B	109.5
C2—C1—C7	119.1 (4)	H15A—C15—H15B	109.5
C6—C1—C7	120.9 (4)	C12—C15—H15C	109.5
C3—C2—C1	120.4 (4)	H15A—C15—H15C	109.5
C3—C2—H2	119.8	H15B—C15—H15C	109.5
C1—C2—H2	119.8	O5—C16—H16A	109.5
C2—C3—C4	120.8 (5)	O5—C16—H16B	109.5
C2—C3—I1	120.1 (4)	H16A—C16—H16B	109.5
C4—C3—I1	119.0 (4)	O5—C16—H16C	109.5
C5—C4—C3	119.0 (5)	H16A—C16—H16C	109.5
C5—C4—H4A	120.5	H16B—C16—H16C	109.5
C3—C4—H4A	120.5	O4—C17—H17A	109.5
C4—C5—C6	122.2 (4)	O4—C17—H17B	109.5
C4—C5—I2	120.3 (4)	H17A—C17—H17B	109.5
C6—C5—I2	117.5 (3)	O4—C17—H17C	109.5
O2—C6—C5	120.1 (4)	H17A—C17—H17C	109.5
O2—C6—C1	122.1 (4)	H17B—C17—H17C	109.5
C5—C6—C1	117.7 (4)		

### Hydrogen-bond geometry (Å, °)

**Table:**

*D*—H···*A*	*D*—H	H···*A*	*D*···*A*	*D*—H···*A*
O4—H4···N2^i^	0.85 (4)	2.03 (5)	2.858 (5)	168 (8)

Symmetry codes: (i) −*x*, −*y*, −*z*+1.
